# 3,5,3′-Triiodothyronine–Loaded Liposomes Inhibit Hepatocarcinogenesis *Via* Inflammation-Associated Macrophages

**DOI:** 10.3389/fonc.2022.877982

**Published:** 2022-05-10

**Authors:** Gangqi Sun, Xiaojuan Hou, Luyao Zhang, Hengyan Zhang, Changchun Shao, Fengwei Li, Chen Zong, Rong Li, Junxia Shi, Xue Yang, Li Zhang

**Affiliations:** ^1^Department of Clinical Pharmacology, The Second Hospital of Anhui Medical University, Hefei, China; ^2^Department of Phase I Clinical Trial, Clinical Research Unit, Changhai Hospital, Naval Medical University, Shanghai, China; ^3^Tumor Immunology and Gene Therapy Center, Third Affiliated Hospital of Second Military Medical University, Shanghai, China; ^4^Department of Tumor Immunity and Metabolism,The National Center for Liver Cancer, Shanghai, China; ^5^School of Pharmacy, Anhui Medical University, Hefei, China; ^6^Department of Oncology, The First Affiliated Hospital of Anhui Medical University, Hefei, China; ^7^Department of Hepatobiliary Surgery, Eastern Hepatobiliary Surgery Hospital, Naval Medical University, Shanghai, China; ^8^Laboratory Zone, Eastern Hepatobiliary Clinical Research Institute, Third Affiliated Hospital of Naval Medical University, Shanghai, China

**Keywords:** 3,5,3′-triiodothyronine, liposome, hepatocarcinogenesis, inflammation, macrophage

## Abstract

**Background:**

Hepatocellular carcinoma (HCC) is inflammation-related cancer. Persistent inflammatory injury of the liver is an important factor mediating the occurrence and development of liver cancer. Hepatic macrophages play an important role in the inflammatory microenvironment, which mediates tumor immune escape, tumor growth, and metastasis. Previous studies have suggested that L-3,5,3-triiodothyronine (T3) can regulate inflammation; however, its use is associated with serious cardiac side effects, and its role in hepatocarcinogenesis remains unclear. In this study, we aimed to develop an effective T3 delivery system with reduced cardiac toxicity and to explore its effects on HCC occurrence.

**Methods:**

T3 liposomes (T3-lipo) were prepared using the thin-film hydration method, and their characteristics, including particle size, polydispersity index, zeta potential, encapsulation efficiency, drug loading, drug release, and stability, were evaluated *in vitro*. We assessed the effect of T3-lipo on hepatocarcinogenesis in diethylnitrosamine (DEN)–induced primary HCC in rats and examined the biodistribution of T3 and T3-lipo by high-performance liquid chromatography–mass spectrometry. Furthermore, we explored the potential molecular mechanism of T3-lipo in hepatocarcinogenesis by immunohistochemistry and immunofluorescence analyses, Bio-Plex assays, real-time polymerase chain reaction analysis, and Western blotting assays.

**Results:**

Compared with T3, T3-lipo had an enhanced inhibitory effect on hepatocarcinogenesis and reduced cardiac side effects in DEN-induced primary HCC in rats. Mechanistically, T3-lipo were absorbed by hepatic macrophages and regulated the secretion of inflammatory cytokines in macrophages by inhibiting inflammatory signaling pathways.

**Conclusions:**

T3-lipo may suppress hepatocarcinogenesis by regulating the inflammatory microenvironment in the liver and reduce the cardiac side effects meanwhile.

## Introduction

Liver cancer has a high incidence and is the second leading cause of cancer-related deaths worldwide (1). Hepatocellular carcinoma (HCC) is the most common type of primary liver cancer and usually occurs at the end stage of chronic liver disease associated with viral hepatitis, alcoholism, and non-alcoholic fatty liver disease (2, 3). These diseases are usually accompanied by chronic hepatitis. Although acute liver inflammation plays a vital and positive role in response to acute infection or liver damage, the effect of chronic liver inflammation may drive the onset and development of HCC (4–6). Collectively, HCC is a typical inflammation-related disorder. It is difficult to cure at advanced stages and is associated with a high risk of recurrence after resection. Therefore, it is essential to better understand the pathogenesis links the inflammation and hepatocarcinogenesis and to explore more effective therapeutic strategies.

The inflammatory microenvironment of the liver involves numerous inflammatory cells and cytokines. Liver macrophages, also known as the Kupffer cells, are abundant in the liver inflammatory microenvironment, playing a key role. During the inflammatory injury stage of the liver, hepatic macrophages aggravate the disorder by secreting inflammatory factors and chemokines, and then, the recruited monocytes further accumulate in the liver and aggravate the inflammatory response (7, 8). Whether inflammation–tumor transformation and postoperative recurrence can be prevented by regulating the inflammatory microenvironment requires further investigation.

L-3,5,3-Triiodothyronine (T3), also known as the thyroid hormone (TH), secreted from the thyroid gland, is the main biologically active form of TH. T3 plays a key role in the endocrine system; controls the metabolism of lipids, proteins, and carbohydrates; and affects neurodevelopment and cardiovascular and brain function (9, 10). Notably, data on the relationship between TH and inflammation are emerging (11). A cohort study showed that low plasma T3 is a strong and independent predictor of death in end-stage renal disease patients, where it may mediate some of the adverse effects of inflammation (12). In addition, exogenous T3 application has shown a positive effect on neurogenesis *via* the regulation of proinflammatory cytokine activity (13). T3 also attenuates renal injury in five of six nephrectomized rats by reducing the rates of oxidative stress, inflammation, apoptosis, and fibrosis in the kidneys (14). T3 has also been reported to regulate inflammation in the liver. In mouse models of concanavalin A (Con A)–induced hepatitis, a derivative of TH, TRIAC, alleviates inflammation by inhibiting the phosphorylation of Akt and MAPKs (15). Perrotta et al. suggested that T3 plays a protective role in inflammation, which is coupled with the modulation of peritoneal macrophage content (16). Moreover, some studies have pointed out that hypothyroidism is highly correlated with the occurrence and development of HCC (17–19), whereas the role and specific mechanism of T3 in hepatocarcinogenesis deserve further exploration. Therefore, whether T3 is involved in hepatocarcinogenesis by regulating the inflammatory state warrants further study.

Nevetheless, T3 administration is accompanied by serious side effects, specifically, cardiac dysfunction, including arrhythmia and increased cardiac output (20, 21). Therefore, an effective drug delivery system with reduced cardiac toxicity must be developed for T3 administration.

Liposomes have been widely used as efficient carrier systems to deliver various therapeutic agents because of their excellent pharmacokinetic characteristics, biocompatibility, and low toxicity (22, 23). After intravenous administration (i.v.), most liposomes are captured by the mononuclear phagocyte system (MPS), specifically, by hepatic macrophages, which account for 80%–90% of the total mononuclear phagocyte population in the body (24–26). Therefore, liposomes are more likely to accumulate in the hepatic macrophages. However, it remains unclear whether T3-loaded liposomes can prevent hepatocarcinogenesis by regulating hepatic macrophages, and any underlying mechanism is unclear.

In this study, we aimed to develop an effective delivery system to reduce cardiac toxicity by using liposomes encapsulating T3. We attempted to explore its regulatory effects on HCC occurrence in diethylnitrosamine (DEN)–induced primary HCC in rats and to determine whether T3-lipo administration reduces the cardiac side effects and further illustrate the potential mechanism underlying this phenomenon.

## Materials and Methods

### Preparation of T3-Lipo

1-Palmitoyl-2-oleoyl-sn-glycero-3-phosphocholine (POPC, 850457P) and dimethyldiocta-decylammonium (DDAB, 890810P) were purchased from Avanti Polar Lipids (Alabaster, USA). 3,3′,5-Triiodo-L-thyronine (T3, T2877) was obtained from Sigma-Aldrich (St. Louis, MO, USA). Trichloromethane (990340AR60) was purchased from Chinasun Specialty Products Co., Ltd (Changshu, China). Methanol (M813895) was purchased from Shanghai Macklin Biochemical Co., Ltd (Shanghai, China).

T3-lipo was prepared using the thin-film hydration method according to previously described methods (27). Briefly, POPC (96.96 µmol, 73.7 mg) and DDAB (0.8 µmol, 0.5 mg) were dispersed in 20 ml of a trichloromethane-methanol mixture (1/1, v/v), and T3 (12 µmol, 8 mg) was dispersed in 5 ml of a mixture of ammonia and methanol (1 ml + 5ml). After mixing these solutions together, a thin lipid film was formed by removing the organic solvent in a rotary evaporator at 45°C and drying under vacuum for 1 h. The lipid film was hydrated with 5 ml of phosphate-buffered saline (PBS, pH 7) at 65°C for 2 h. Liposomes were sonicated four times for 1 min each time using an ultrasonic cell disruptor (SCIENTZ, Ningbo, China). The mixture was centrifuged at 15,000 rpm for 1 h to remove unencapsulated T3. The liposomes were then filtered through a 0.2-μm polycarbonate membrane six times in a gas-tight syringe (610017-1Ea, Avanti, USA).

Liposomes labeled with fluorescent dyes (Rho-T3-lipo) were prepared for cellular uptake analysis by adding lipophilic Rho-PE (0.12 µmol, 0.154 mg) in the lipid mixture, following the same preparation method.

### Characterization of T3-Lipo

The particle size, polydispersity index (PDI), and zeta potential of T3-lipo were measured by dynamic light scattering (Malvern Instruments, Malvern). Encapsulation efficiency (EE) and drug loading (DL) values of T3-lipo were measured by high-performance liquid chromatography–mass spectrometry (HPLC) (Agilent1100, Agilent, USA) at a wavelength of 305 nm, where the column was Agilent ZORBAX SB-C18(5μm, 4.6 × 150 mm), and the column temperature was 25°C. The mobile phase involved a mixture of acetonitrile-acetic acid (1,000:2, v/v) and water–acetic acid (1,000:2, v/v), delivered at a flow rate of 1 ml/min.

The release of T3 from T3-lipo was determined using the dialysis method. The release medium was PBS (pH 7.4), containing 1% Tween 80. Briefly, 1 ml of the sample was placed into a pre-wetted RC dialysis bag (Molecular Weight Cut Off 3.5 kDa), sealed, and immersed in 400 ml of a release solution. The medium was incubated at 37°C with stirring at 100 rpm. At predetermined time intervals (0, 0.5, 1, 2, 4, 8, 12, 24, 48, and 72 h), 4 ml of the medium was withdrawn and replaced with an equal amount of fresh medium. The cumulative amount of T3 in the samples was determined using HPLC at a wavelength of 305 nm. All measurements were performed in triplicate.

Liposome stability under *in vitro* storage conditions is important for both *in vitro* and *in vivo* biomedical applications. The T3-lipo samples were stored at 4°C for 1, 3, 5, and 7 days after preparation, and particle size, PDI, EE, and DL values were measured to evaluate liposome stability.

### Animal Models and Treatments

Male SD rats (180–200 g) were obtained from the Shanghai Laboratory Animal Center (Shanghai, China) and maintained under pathogen-free conditions at the Laboratory Animal Center of the Second Military Medical University (Shanghai, China). All animal protocols were approved by the Animal Care Committee of the Second Military Medical University.

All animals were fed adaptively for 1 week and then received 0.01% DEN (Sigma-Aldrich, Shanghai, China) through drinking water. Two experimental protocols were used to study hepatocarcinogenesis in rats.

On the basis of the previous research on T3 administration schemes (13, 14, 28–31), in the early stage of the study, we have explored the effects of different dosages (0.1~10 mg/kg) and different ways of administration (tail vein injection/spleen injection) of T3 and T3-lipo on hepatocarcinogenesis in DEN-induced primary HCC rat models (data not shown). According to the results of our pre-study, here, we present the results of two modes of T3 and T3-lipo administration, that is, low-dose high-frequency administration and high-dose low-frequency administration. The two experimental protocols are as follows.

Experimental protocol 1: Rats exposed to DEN for 8 weeks were randomly divided into three treatment groups (four rats in each group) and injected with saline, T3, or T3-lipo through the spleen with a T3 dose of 0.5 mg/kg once a week. The rats were sacrificed at 16 weeks in DEN-induced models, after the total of four treatment cycles.

Experimental protocol 2: Rats exposed to DEN for 10 weeks were randomly divided into three groups (saline, T3, and T3-lipo). The rats (n = 4) were given intravenous injections of saline, T3, or T3-lipo *via* the tail vein only once with a T3 dose of 5 mg/kg. The rats were sacrificed 0.5 h, 1 week, and 6 weeks thereafter for the immunofluorescence (IF) assay, and to explore the mechanism of and to describe hepatocarcinogenesis, respectively.

The evaluation of the number and max volume of tumors was determined by counting the number of visible tumors and measuring the size (the longest and shortest diameters) of the largest tumor with a caliper (32). Calculation formula of max volume of tumor: V(mm^3^) = a × b^2^/2 (a, the longest diameters; b, the shortest diameters).

### Biodistribution Assay

Rats exposed to DEN for 10 weeks were randomly divided into two groups (n = 3). The rats were fasted overnight before treatment administration but allowed free access to water. T3 and T3-lipo were given intravenously at a dose of 5 mg/kg. Blood samples were obtained from the inferior vena cava at 10 min, 0.5, 4, 8, and 24 h after administration, and left standing for 30 min before being centrifuged at 3,000 rpm for 10 min at 4°C. Serum was separated and stored at −80°C until analysis. The heart, liver, spleen, lung, and kidney were immediately collected and washed in ice-cold saline, blotted with paper towel to remove excess fluid, weighed, and stored at −80°C until analysis. All samples were assessed for T3 concentration using high-performance liquid chromatography–mass spectrometry.

### Cell Line and Treatment

NR8383 (rat alveolar macrophage cell line) cells were obtained from The Chinese Academy of Sciences cell bank, incubated in Ham’s F12K medium (Gibco) with 20% FBS and 1% penicillin/streptomycin, in a humidified atmosphere with 5% CO_2_ at 37°C.

To establish lipopolysaccharides (LPS)–induced inflammatory injury models, NR8383 cells were stimulated with LPS (100 ng/ml) (Sigma, Saint Louis, MO, USA), followed by supplemented with T3 (500 nM/1 µM). After 12 h of treatment, RNA was extracted for PCR analysis. The conditioned culture supernatant and the protein of cells were collected after 24 h of treatment for Bio-Plex and Western Blotting analysis

### Primary Peritoneal Macrophages Isolation and Culture

Peritoneal macrophages were isolated from SD rats exposed to DEN for 10 weeks *via* peritoneal lavage. Briefly, peritoneal macrophages were isolated after the peritoneal cavity was washed with PBS for 10 min. The peritoneal fluid was gently collected into tubes and centrifuged at 1,000 rpm for 10 min. The pellets containing cells were washed and resuspended in RPMI 1640 medium with 10% FBS for 4 h in a humidified atmosphere with 5% CO_2_ at 37°C.

For the cellular uptake assay of T3-lipo *in vitro*, peritoneal macrophages were plated in a 24-well plate at 5 × 10^5^ cells per well and cultured with Rho-T3-lipo at a concentration of 10 µg/ml for 10 min, stained with (4',6-diamidino-2-phenylindole) (Thermo Fisher Scientific, USA), and scanned using a confocal microscope (Leica TCS SP2; Wetzlar, Germany).

### Primary Hepatic Macrophage Isolation and Culture

Primary hepatic macrophages were isolated from SD rats that received DEN for 11 weeks, as described in Experimental Protocol 2. The rats were anesthetized with 10% chloral hydrate and perfused with calcium- and magnesium-free PBS through the portal vein. After the liver was fully expanded, the inferior vena cava was cut off to remove liquid, and the liver was perfused with 15 ml of RPMI 1640 medium containing 0.1% collagenase IV and cut into small pieces. The liver tissue was dispersed with 10 ml of 0.1% collagenase IV, incubated in a water bath at 37°C for 30 min, and then passed through 70-µm filter membrane to remove the undigested tissue. The cells were suspended in 10 ml of RPMI 1640 medium and centrifuged at 600 rpm for 10 min to isolate hepatocytes. The resulting supernatant was centrifuged at 1,500 rpm for 5 min, and the cell pellet was resuspended in 4 ml of (Hank's Balanced Salt Solution). Equal volumes of 60% and 30% separation solutions were added, and the cell suspension was placed on the top layer. The mixture was centrifuged at 2,000 rpm for 15 min, and the middle layer was collected. The collection was washed with 5 ml of HBSS and centrifuged at 1,500 rpm for 5 min to obtain hepatic macrophages. The hepatic macrophages were incubated in RPMI 1640 medium with 10% FBS and 1% penicillin/streptomycin in a humidified atmosphere with 5% CO_2_ at 37°C.

### The Liver Function, Cardiac Function and Bio-Plex Assays

Serum alanine aminotransferase (ALT), aspartate aminotransferase (AST), creatine kinase (CK), and creatine kinase isoenzyme (CK-MB) levels were measured by Fuji DRICHEM 55500V (Fuji Medical Systems, Japan) according to the manufacturer’s protocols. Concentrations of inflammatory cytokines from rat serum and conditioned culture supernatant of NR8383 cells were measured by the Bio-Plex Pro Rat Cytokine, Chemokine, and Growth Factor Assays (Bio-Rad Laboratories, Hercules, CA, USA) according to the manufacturer’s instructions.

### Enzyme-Linked Immunosorbent Assay

Serum Troponin I (Tn-I) was measured by enzyme-linked immunosorbent assay (ELISA). The rat Tn-I ELISA kit was purchased by Kenuodibio (Quanzhou, China). The procedure for the determination of Tn-I in rat serum was detected according to the manufacturer’s protocols.

### RNA Extraction and Real-Time Polymerase Chain Reaction

Total RNA was extracted from cells or liver tissue using TRIzol reagent (Invitrogen, USA) according to the manufacturer’s protocols. cDNA was performed by a Bestar™ qPCR RT Kit (DBI, Germany), and real-time polymerase chain reaction (PCR) was detected by Bestar^®^ SybrGreen qPCR mastermix (DBI, Germany) to analyze the relative RNA expression levels of the targets. The primer sequences were displayed in **Table S2**.

### Western Blotting Assay

Protein samples were collected from primary hepatic macrophages and NR8383 cells, respectively. Cells were lysed using (Radio Immunoprecipitation Assay) lysis buffer conjugated with protease and phosphatase inhibitor cocktail (Beyotime, China) to separate the total proteins. After quantification by bicinchoninic acid (BCA) protein assay kits (Beyotime, China), equal amount of protein was electrophoresed *via* 4%–12% Bis-Tris SurePAGE gels (GenScript, China), followed by transfer onto (poly(1,1-difluoroethylene)) membranes (Merck Millipore, Germany). After blocking in 5% BSA for 1.5 h, the membranes were then incubated with diluted primary antibodies overnight at 4°C. Thereafter, the membranes were further incubated with the secondary antibody for 1.5 h and then were visualized using enhanced chemiluminescence detection reagents (GE Healthcare, USA). The antibodies used in the assay were in the following: anti-p38 (1:1,000; CST, USA), anti–phospho-p38 (1:1,000; CST, USA), anti-(extracellular regulated protein kinases1/2) (1:1,000; (Cell Signaling Technology), USA), anti–phospho-ERK1/2 (1:1,000; CST, USA), anti-(c-Jun N-terminal kinase) (1:1,000; CST, USA), anti–phospho-JNK (1:1,000; CST, USA), anti–(nuclear factor kappa-B) p65 (1:1,000; CST, USA), anti–phospho-NF-κB p65 (1:1,000; CST, USA), anti-IκBα (1:1,000; CST, USA), anti–phospho-(inhibitor of NF-κB) (1:1,000; CST, USA), anti–β-actin (1:4,000; Bioworld, USA), anti–ɑ-MyHC (1:2,000; Abcam, UK), and anti-SERCA2 (1:1,000; Abcam, UK) antibodies.

### Histological Examination

Paraffin-embedded liver samples were cut into 5-μm sections for hematoxylin and eosin (H&E). Image-Pro Plus software version 6.2 (Media Cybernetics Inc., Bethesda, MD, USA) was used to analyze the images.

### Immunohistochemistry and Immunofluorescence Analysis

The immunohistochemistry (IHC) analysis was performed according to the manufacturer’s protocols. The used antibodies were as follows: anti-CD68 (1:200; Abcam, UK), anti-iNOS (1:100; Abcam, UK), and anti-CD163 (1:500; Abcam, UK). The density of positive immunostaining was evaluated by Image-Pro Plus software version 6.2. The IOD (integrated optical density) was measured to analyze the mean density, which was calculated as IOD per total area of each image. For IF analysis, frozen sections of the liver tissues were cut into 8-μm sections and stained with the indicated antibodies: anti-CD68 (1:200; Abcam, UK). The nuclei were stained with DAPI (Thermo Fisher Scientific, USA). The sections were subsequently scanned using a confocal microscope (Leica TCS SP2; Wetzlar, Germany).

### Statistical Analysis

All experiments were performed at least three times. The data were presented as the mean ± standard deviation. Analysis of significance was done by GraphPad Prism 8 (GraphPad Software Inc., CA). Significance between groups was performed using one-way ANOVA and Student’s *t*-test. P-values less than 0.05 indicated statistically significant.

## Results

### Preparation and Characterization of T3-Lipo

**Figure 1:**
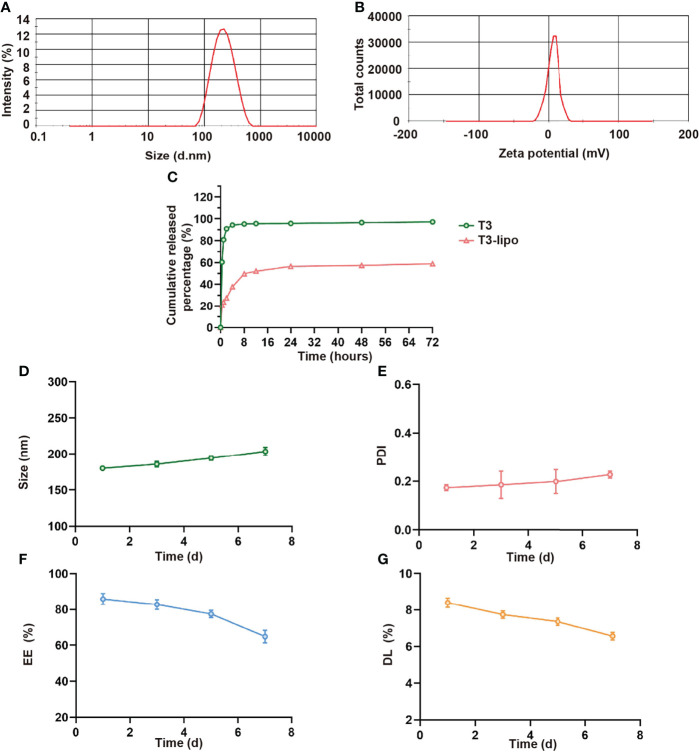
Characterization of T3-lipo. **(A)** Size distribution of T3-lipo. **(B)** Zeta potential distribution of T3-lipo. **(C)** *In vitro* release profiles of T3 from T3-lipo in phosphate-buffered saline (PBS, pH = 7.4) over 72 h. **(D–G)** Stability of T3-lipo in particle size, polydispersity index (PDI), encapsulation efficiency (EE), and drug loading (DL) values over 7 days at 4°C. Mean ± SD values were obtained from at least three independent experiments performed in triplicate.

T3-lipo characteristics, including particle size and Zeta potential, are presented in **Figures 1A, B**. The average particle size of T3-lipo was 199.73 nm with a narrow size distribution (PDI < 0.3), Zeta potential of 4.84mV, and surface charges that were nearly neutral. The EE of T3-lipo was 88.89 ± 0.059%, and DL was 8.67 ± 0.0058%. The characterization data for Rho-T3-lipo are shown in **Table S1**.

To investigate whether the encapsulated T3 was released from T3-lipo at a slow and sustained rate, *in vitro* drug release profiles were determined (**Figure 1C**). The release rate was divided into two stages within 72 h. For free drugs, T3 was released at a burst rate within the initial 4 h, followed by a sustained release for 72 h, whereas 50% of T3-lipo was released at a relatively slow rate within the initial 8 h. The cumulative release rate of liposomes within 72 h was less than 60%, whereas the cumulative release rate of T3 was more than 95%, indicating that T3 release was delayed after liposome loading.

Stability is an important parameter of drug-loaded liposomes. To assess the stability of T3-lipo, the particle size, PDI, EE, and DL values were measured in PBS (pH 7.4) at 4°C for 7 days. There were no significant changes in these parameters, indicating that the prepared T3-lipo was stable at 4°C during storage (**Figures 1D–G**).

### T3-Lipo Inhibits Hepatocarcinogenesis With Reduced Cardiotoxicity

The experimental design is shown in **Figure S1A**. Animals exposed to DEN for 8 weeks were injected with T3 or T3-lipo at a dose of 0.5 mg/kg once a week through spleen injection and four times totally. The visceral views of the liver are shown in **Figure S1B**. Animals treated with saline displayed multiple large tumor nodules, whereas the livers of T3-exposed rats were characterized by few macroscopic tumor nodules. Compared with T3, T3-lipo exhibited a better inhibitory effect on hepatocarcinogenesis. The animals treated with T3 displayed a significant reduction in the incidence of HCC, as indicated by the significantly reduced count and volume of maximum tumor nodules. T3-lipo performed relatively well at the above indexes (**Figures S1C–E**). Moreover, the effect of T3 and T3-lipo was evaluated by H&E staining (**Figure S1F**). Tissue injury and pathological structural disorders of the liver were significantly improved after treatment with T3; however, the effects of T3-lipo were greater than those of T3. Notably, T3 treatment induced high levels of CK and CK-MB in serum, whereas T3-lipo treatment relieved it (**Figures S1G, H**). The phenomenon implied that liposomes loaded T3 may reduce the cardiotoxicity compared to T3.

**Figure 2:**
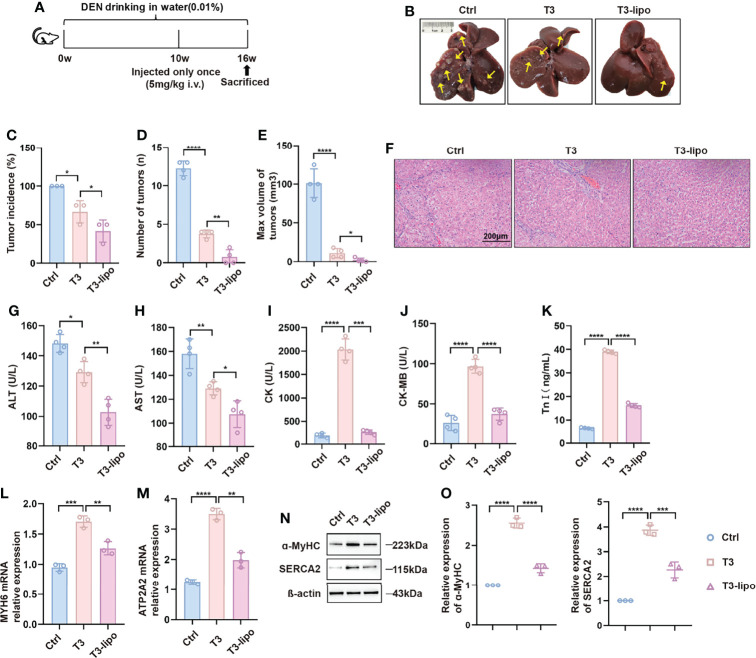
T3-lipo inhibits hepatocarcinogenesis with reduced cardiotoxicity in DEN-induced primary HCC rat models. **(A)** Experimental design. **(B)** Representative upper and visceral views of livers from rats treated with saline, T3, and T3-lipo at a dose of 5 mg/kg *via* the tail vein. **(C)** Tumor incidence (%). **(D)** Number of tumors (n). **(E)** Max volume of tumors (mm³). V(mm^3^) = a × b^2^/2 (a, the longest diameters; b, the shortest diameters). **(F)** H&E staining of liver tissues. **(G)** Serum levels of ALT(U/L). **(H)** Serum levels of AST(U/L). **(I)** Serum levels of CK(U/L). **(J)** Serum levels of CK-MB(U/L). **(K)** Serum levels of Tn-I (ng/ml). n = 4 rats per group. Three independent experiments were performed for all data. **(L)** RT-PCR analysis of MYH6 in heart tissues. **(M)** RT-PCR analysis of ATP2A2 in heart tissues. **(N)** Western blotting assay of ɑ-MyHC and SERCA2 in heart tissues. **(O)** Densitometry of ɑ-MyHC and SERCA2 was performed followed by normalization. Mean ± SD values were obtained from at least three independent experiments performed in triplicate. Differences were analyzed using one-way ANOVA (*p < 0.05, **p < 0.01, ***p < 0.001, and ****p < 0.0001).

Furthermore, the administration scheme was optimized considering the limitations of the injection method. Animals exposed to the experimental design shown in **Figure 2A** were injected with T3 or T3-lipo at a dose of 5 mg/kg *via* the tail vein 10 weeks after DEN administration and sacrificed 6 weeks thereafter. As expected, a single megadose could also inhibit hepatocarcinogenesis, and T3-lipo exhibited an enhanced inhibitory effect on hepatocarcinogenesis (**Figures 2B–E**). H&E staining showed that the pathological structure of the liver was effectively improved after administration (**Figure 2F**). In addition, the treatment groups presented lower serum levels of ALT and AST at 11 weeks after DEN administration, particularly in the T3-lipo treatment groups (**Figures 2G, H**). Combined with the existing literature on T3 and inflammation (13–15), the decreased levels of ALT and AST may be accounted for the improved inflammatory microenvironment in the liver by T3 or T3-lipo, thereby the death of the hepatocytes was inhibited. Data from the H&E staining assay suggested that poor liver function was significantly improved by both T3 and T3-lipo. Meanwhile, the serum levels of CK, CK-MB, and Tn-I were high in T3 treatment groups, and T3-lipo induced lower levels compared to T3 (**Figures 2I–K**). These cardiac function indicators implied that T3 may cause myocardial damage, whereas T3-lipo alleviated it. Further, we analyzed the expression of T3-target genes (MYH6 and ATP2A2) in heart tissues by RT-PCR and Western blotting assays. The results showed that the mRNA transcription levels of MYH6 and ATP2A2 were increased in T3 treatment groups compared to the control groups, and T3-lipo reduced the increase (**Figures 2L, M**). The corresponding protein levels of T3-target genes (ɑ-MyHC and SERCA2) were evaluated by a Western blotting assay, and the results were consistent with the data presented in RT-PCR (**Figures 2N, O**). These findings may be accounted for by the application of liposomes, which increase the effective delivery of T3 to the liver and reduce the non-specific uptake by other tissues, leading to stronger inhibition of hepatocarcinogenesis and lighter cardiotoxicity.

To observe the histological characteristics of other major organs after treatment, different ways of administration (saline, T3, and T3-lipo) were performed at a dose of 5 mg/kg *via* the tail vein 10 weeks after DEN administration. The rats were sacrificed at 11 weeks after DEN administration. The experimental design is shown in **Figure S2A**. As shown in **Figure S2B**, massive inflammatory cell infiltration was observed in the T3 treatment groups in heart tissues, and the phenomenon was relieved in T3-lipo treatment groups. We found a slight increase of thickness of interalveolar septa after T3-lipo treatment in the lung, which may be attributed to the accumulation of liposomes in dedicated host filtration organs (24). No obvious histopathological changes were observed in spleen and kidney tissues.

### T3-Lipo Attenuate Liver Inflammation

**Figure 3:**
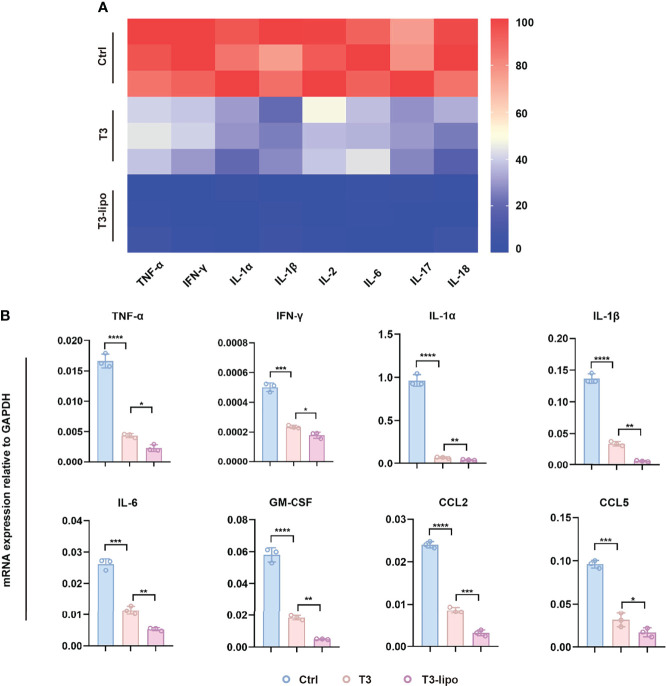
T3-lipo attenuates liver inflammation in DEN-induced primary HCC rat models. **(A)** Bio-Plex assays of inflammatory cytokines (TNF-α, IFN-γ, IL-1α, IL-1β, IL-2, IL-6, IL-17, and IL-18) in serum. **(B)** RT-PCR analysis of TNF-α, IFN-γ, IL-1α, IL-1β, IL-6, GM-CSF, CCL2, and CCL5 mRNA in livers treated with saline, T3, or T3-lipo in DEN-induced rat models. Mean ± SD values were obtained from at least three independent experiments performed in triplicate. Differences were analyzed using One-way ANOVA (*p < 0.05, **p < 0.01, ***p < 0.001, and ****p < 0.0001).

Considering the enhanced inhibitory effect on hepatocarcinogenesis that was observed in experimental protocol 2 and the limitation of injection through the spleen, we chose the samples of experimental protocol 2 for the following mechanism research. To verify the effect of T3 on inflammation, we analyzed the serum levels of pro-inflammatory cytokines. Indeed, inflammatory cytokines were significantly downregulated after treatment, particularly in the T3-lipo treatment groups (**Figure 3A**). In addition, we examined the mRNA transcription levels of the inflammatory cytokines in liver tissues by real-time PCR (RT-PCR) analysis, showing that T3 and T3-lipo reduced the levels of gene expression of inflammatory cytokines, particularly in the T3-lipo treatment groups (**Figure 3B**).

It is well known that HCC is a typical inflammation-related disorder (4–6). Hepatic macrophages play a key role in the inflammatory microenvironment in the liver (7, 8). Focusing on the effect of the inflammatory microenvironment on hepatocarcinogenesis, we performed IHC assays to analyze the possible effect on the polarization of macrophages in peritumoral tissues after T3 or T3-lipo treatment. As shown in **Figure S3**, the expression of the total macrophage marker CD68 and the pro-inflammatory M1 macrophages marker iNOS were all decreased, whereas the expression of CD163, a marker of M2 macrophages, remained unchanged.

### T3-Lipo Are Absorbed by Hepatic Macrophages and Enriched in Liver

**Figure 4:**
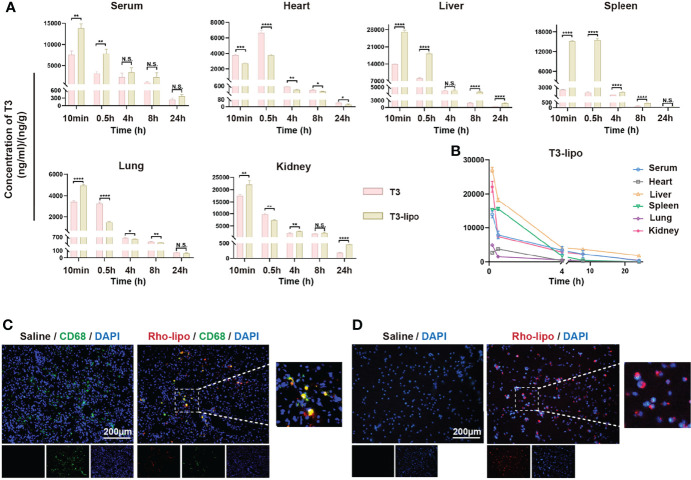
T3-lipo are taken by hepatic macrophages and enriched in the liver. **(A)** Concentration–time profiles of T3 in serum, heart, liver, spleen, lung, and kidney after i.v. administration of T3 and T3-lipo at a dose of 5 mg/kg in DEN-induced primary HCC rat models. **(B)** Concentration–time profiles of T3 in serum, heart, liver, spleen, lung, kidney after i.v. injected with T3-lipo at a dose of 5 mg/kg in DEN-induced primary HCC rat models. n = 3 rats per group. Three independent experiments were performed for all data. Differences were analyzed using Student’s *t*-test (*p < 0.05, **p < 0.01, ***p < 0.001, and ****p < 0.0001; N.S., not significant). **(C)** Frozen section immunofluorescence of rat liver tissues after intravenous injection of saline or Rho-T3 liposome (5 mg/kg) for 30 min in DEN-induced primary HCC rat models. **(D)** The fluorescence microscopy images of rat peritoneal macrophages incubated with saline or Rho-T3-lipo (10 ug/ml) for 30 min at 37°C.

After observing hepatocarcinogenesis and inflammation inhibition by T3-lipo, we examined the enrichment capacity of liposomes in the liver by a biodistribution assay. The biodistribution profiles of T3 and T3-lipo in DEN-induced rats following intravenous administration are shown in **Figure 4A**. The drug concentration–time presentation of T3-lipo varied considerably from that of T3. T3 treatment groups exhibited a relatively rapid decline in blood drug levels, alongside an increased distribution in the heart, compared to those observed in the T3-lipo groups; these findings were consistent with those of rat serum cardiac function *in vivo* analysis. The T3-lipo treatment groups displayed higher T3 levels in the liver, spleen, and kidney, which may be attributed to the biological features of the nanoparticles. T3-lipo accumulated primarily in the liver rather than in the other organs or serum (**Figure 4B**).

After injection, nanoparticles are easily captured by the reticuloendothelial system, which comprises macrophages and monocytes, liver Kupffer cells, lymphatic vessels, and the spleen, which remove foreign material from the body (24).

Thus, we determined whether hepatic macrophages absorbed T3-lipo. An IF assay of rat liver tissues after intravenous injection of saline or Rho-T3-lipo was performed, and red fluorescence of Rho-T3-lipo in hepatic macrophages was observed in liver tissues treated with Rho-T3-lipo (**Figure 4C**). Meanwhile, we extracted peritoneal macrophages from DEN-induced rats for 10 weeks, revealing that the rat primary peritoneal macrophages phagocytosed T3-lipo after culturing with Rho-T3-lipo (**Figure 4D**).

### T3-Lipo Inhibit MAPK and NF-κB Signaling Pathways in Macrophages *In Vivo* and *In Vitro*

**Figure 5:**
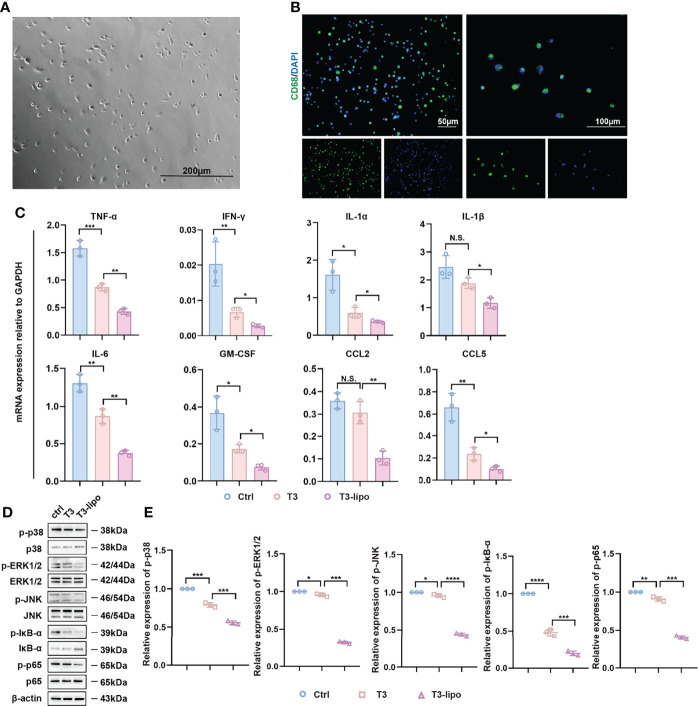
Anti-inflammatory effect of T3-lipo on hepatic macrophages *in vivo*. **(A)** The image of primary hepatic macrophages by microscope. **(B)** The immunofluorescence image of staining for CD68. **(C)** RT-PCR analysis of TNF-α, IFN-γ, IL-1α, IL-1β, IL-6, GM-CSF, CCL2, and CCL5 mRNA in hepatic macrophages of DEN-induced rats treated with saline, T3 or T3-lipo. **(D)** Western blotting assay of MAPK and NF-κB signaling pathways in hepatic macrophages of DEN-induced rats treated with saline, T3, or T3-lipo. **(E)** Densitometry of p-p38, p-ERK1/2, p-JNK, p-IĸB-ɑ, and p-p65 was performed followed by normalization. Mean ± SD values were obtained from at least three independent experiments performed in triplicate. Differences were analyzed using One-way ANOVA (*p < 0.05, **p < 0.01, ***p < 0.001, and ****p < 0.0001; N.S., not significant).

We isolated primary hepatic macrophages from rats that received DEN for 11 weeks (Experimental Protocol 2). After 2 h of incubation, the cells attached to the plate surface. Rinse dishes with PBS to remove non-adherent cells. We observed the cell viability by microscope. As shown in **Figure 5A**, the cells exhibited round nuclei with an irregular outline, which resembles the shape of macrophages reported in the previous literature (33). Next, we performed the IF assay with CD68 for cell identification (**Figure 5B**). At the mRNA level, the expression of inflammatory cytokines, including TNF-α and IFN-γ, was significantly decreased (**Figure 5C**).

In addition, MAPK and NF-κB signaling pathways regulate inflammation (34, 35). Several studies have demonstrated the regulatory role of MAPK and NF-κB signaling pathways in macrophages inflammation (36–38). To assess whether T3 and T3-lipo may regulate inflammatory levels through the MAPK and NF-κB signaling pathways, we performed a Western blotting assay. The phosphorylation of p38, ERK1/2, JNK, p65, and IκB-α was significantly decreased in hepatic macrophages from rats treated with T3 or T3-lipo; the impact of T3-lipo was greater than that of T3. Meanwhile, the levels of p38, ERK1/2, JNK, and p65 remained unchanged, whereas the level of IκB-α was increased by T3 and T3-lipo; the impact of T3-lipo was greater than that of T3 (**Figures 5D, E**).

**Figure 6:**
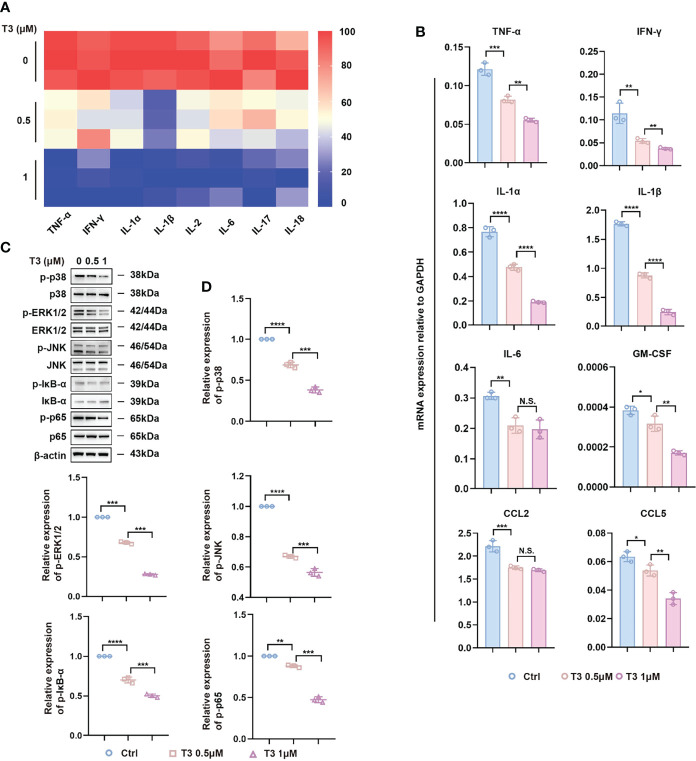
Anti-inflammatory effect of T3 in NR8383 cells *in vitro.* **(A)** TNF-α, IFN-γ, IL-1α, IL-1β, IL-2, IL-6, IL-17, and IL-18 protein levels were determined in the cell supernatants by Bio-Plex. **(B)** RT-PCR analysis of TNF-α, IFN-γ, IL-1α, IL-1β, IL-6, GM-CSF, CCL2, and CCL5 mRNA in NR8383 cells treated with 500 nM or 1 µM T3. **(C)** Western blotting assay of MAPK and NF-κB signaling pathways in NR8383 cells treated with 500 nM or 1 µM T3. **(D)** Densitometry of p-p38, p-ERK1/2, p-JNK, p-IĸB-ɑ, and p-p65 was performed followed by normalization. Mean ± SD values were obtained from at least three independent experiments performed in triplicate. Differences were analyzed using one-way ANOVA (*p < 0.05, **p < 0.01, ***p < 0.001, and ****p < 0.0001; N.S., not significant).

To further evaluate the anti-inflammatory potential of T3 *in vitro*, we established an LPS-induced inflammatory injury model using NR8383 cells. It is well known that liposomes are engulfed into cells *via* phagocytosis and/or endocytosis (39), which makes no difference to the signaling pathways of the mechanism. Therefore, we explored the impact of different concentrations of T3 (0.5 μM, 1 μM) on MAPK and NF-κB signaling pathways. First, we analyzed the levels of pro-inflammatory cytokines in the conditioned culture supernatants and found that LPS induced the expression of pro-inflammatory cytokines, whereas the levels of pro-inflammatory cytokines were downregulated by T3 (**Figure 6A**). In addition, we evaluated the mRNA expression levels of inflammatory cytokines by RT-PCR analysis, showing that T3 decreased the expression of pro-inflammatory cytokines (**Figure 6B**). Consistent with the negative regulation of the MAPK and NF-κB signaling pathways *in vivo*, T3 prevented the activation of the MAPK and NF-κB signaling pathways in NR8383 cells (**Figures 6C, D**).

## Discussion

There is growing evidence that T3 is implicated in the occurrence and development of HCC. Two case-control studies highlighted hypothyroidism as a risk factor for HCC (40, 41). However, in subsequent studies, the specific role and mechanism of T3 in HCC have been controversial. Some studies indicated that T3 plays a suppressive role in the development of HCC (42–44), whereas others reported a promotive role of T3 in HCC (45, 46), involving various cellular processes, such as cell differentiation, metabolism, apoptosis, and angiogenesis. Therefore, the mechanisms underlying the regulatory effects of T3 in HCC onset and progression remain unclear.

Furthermore, T3 administration is associated with serious cardiac side effects, including tachycardia, arrhythmia, and heart failure (20, 21). Some potential mechanisms linking the two conditions are dyslipidemia, endothelial dysfunction, blood pressure changes, and direct effects of THs on the myocardium (21). Therefore, it is necessary to develop a T3 delivery system that reduces the distribution of T3 in extrahepatic tissues, especially heart tissues.

In this study, we used liposomes as drug carriers to encapsulate T3. We first prepared T3-lipo using a thin-film hydration method and fully characterized it. We found that the T3-lipo particle size had a normal distribution, and the PDI was below 0.3, indicating that the particle size distribution was uniform and relatively narrow. The EE and DL values of T3-lipo were considerable. *In vitro* drug-releasing and stability assays showed that the drug release from T3-lipo occurred at a slow, sustained rate, and the T3-lipo preparation was stable at 4°C throughout the monitored storage period. Next, we established DEN-induced primary HCC rat models to study the role of T3-lipo in HCC occurrence. We observed that both T3 and T3-lipo inhibited hepatocarcinogenesis. Interestingly, the T3-lipo treatment had a higher inhibitory efficiency and lower cardiac toxicity than the T3 treatment. We found that the expression levels of T3-target genes in cardiomyocytes, including MYH6 and ATP2A2, were significantly downregulated in T3-lipo treatment groups compared with those in the T3 groups. The T3-lipo treatment was also associated with reduced serum levels of CK, CK-MB, and Tn-I.

Previous reports indicate that most liposomes may be captured by the MPS, which is present in dedicated host filtration organs (i.e., liver, spleen, and kidney) (24). In our *in vivo* study, the distribution of T3-lipo was higher in dedicated host filtration organs (i.e., liver, spleen, and kidney) and lower in heart tissues compared with that of T3, which could explain the reduced cardiac toxicity in DEN-induced primary HCC rat models after T3-lipo administration. Thus, an advantage of using liposomes as drug carriers was the improved distribution of T3 *in vivo*. In addition, H&E staining of tissue samples revealed a slight increase in the thickness of interalveolar septa after T3-lipo treatment in the lung. Further studies should aim to design ligand-modified T3 liposomes to increase the targeting in the liver and reduce the distribution of T3-lipo in the lung.

Several studies showed that HCC is a typical inflammation-related disorder, and persistent inflammatory injury of the liver drives the occurrence and progression of HCC (47, 48). In our study, inflammatory cytokines were significantly downregulated after treatment, especially in the T3-lipo treatment groups. Thus, T3-lipo improved the liver inflammatory microenvironment, which may represent evidence of tumor suppression.

Numerous immune cells (i.e., hepatic macrophages, dendritic cells, neutrophils, and natural killer cells) and inflammatory cytokines (i.e., TNF-α, IFN-γ, CCL2, and CCL5) constitute the inflammatory microenvironment of the liver and jointly motivate tumor immune evasion, tumor progression, and metastasis (49, 50). As a heterogeneous cell population, hepatic macrophages play a central role in the inflammatory microenvironment of the liver and participate in coordinating immune response, removing pathogens, and antigen presentation (51). In 2002, Mantovani et al. described the heterogeneity and plasticity of macrophages when exposed to the external microenvironment (52). Subsequently, an increasing number of studies have shown that M2 macrophages, a key component of inflammatory circuits that promote tumor progression and metastasis, participate in circuits that regulate tumor growth and progression, adaptive immunity, stroma formation, and angiogenesis (53, 54). Our results indicated that T3-lipo affected the polarization of macrophages; specifically, the number of pro-inflammatory M1 macrophages decreased and that of M2 macrophages remained unchanged. Considering the deficiency of experimental methods that we performed, further evidence needs to investigate using serial sections for more convincing staining. Moreover, a series of inflammatory factors and chemokines were downregulated. Thus, T3-lipo evidently attenuated the inflammatory microenvironment of the liver.

A few previous studies have reported the regulatory effects of T3 on the inflammatory response and phagocytosis of macrophages (55, 56). Perrotta et al. suggested that T3 induced the pro-inflammatory phenotype of macrophages, but T3 was also associated with a significantly improved survival rate in LPS-induced endotoxemia mice (16). In general, the specific effects of T3 on macrophages remain poorly understood and deserve further investigation. In the present study, IF analysis revealed the association between T3-lipo and hepatic macrophages. When we isolated the primary rat liver macrophages, we found that T3-lipo inhibited MAPK and NF-κB signaling pathways in the macrophages and negatively regulated the inflammatory response. Thus, the above observations were consistent with our *in vitro* analysis results.

## Conclusion

In summary, T3-lipo were prepared and their inhibitory and toxicity effects were evaluated in hepatocarcinogenesis in rat models of DEN-induced HCC. T3-lipo regulated the levels of inflammatory cytokines in hepatic macrophages and improved the inflammatory microenvironment in liver. Thus, T3-lipo may be a candidate intervention in HCC onset or recurrence after resection.

## Data Availability Statement

The raw data supporting the conclusions of this article will be made available by the authors, without undue reservation.

## Ethics Statement

The animal study was reviewed and approved by the Second Military Medical University Animal Care Committee.

## Author Contributions

LZ and XY conceived and designed the research work. GQS and XJH performed the experiments and acquired the data. LYZ and HYZ assisted to complete the experiments. CCS, FWL, CZ, RL, and JXS interpreted and analyzed the data. All authors contributed to the article and approved the submitted version.

## Funding

This project was supported by the National Key R&D Program of China (Grant Nos. 2018YFA0107500 and 2017YFA0504503), National Natural Science Foundation of China (Grant Nos. 81872243, 81972599, 82073032, 82073037, and 82173276), the municipal hospital medical‐enterprise collaborative clinical trial management project of Shanghai Shenkang Hospital Development Center (20CR4008B), the guaranteed discipline promotion project of Changhai Hospital (2020YBZ011), and Shanghai Municipal Health Bureau (Grant No. 20214Y0013).

## Conflict of Interest

The authors declare that the research was conducted in the absence of any commercial or financial relationships that could be construed as a potential conflict of interest.

## Publisher’s Note

All claims expressed in this article are solely those of the authors and do not necessarily represent those of their affiliated organizations, or those of the publisher, the editors and the reviewers. Any product that may be evaluated in this article, or claim that may be made by its manufacturer, is not guaranteed or endorsed by the publisher.
