# Supplementary material for: 3,5,3′-Triiodothyronine–Loaded Liposomes Inhibit Hepatocarcinogenesis Via Inflammation-Associated Macrophages
Source: Front Oncol. 2022 May 10;12:877982. doi: 10.3389/fonc.2022.877982 (PMC9135096; doi:10.3389/fonc.2022.877982)

Supplementary Material

# Supplementary Data

**Table S2.** Real-time PCR primers

| Primers Names | Sequence 5' to 3' |
| --- | --- |
| IL-6 F | CTGGCAATATGAATGTTGAAAC |
| IL-6 R | AGAAACCATCTGGCTAGGTAAG |
| IL-1β F | CACCTCTCAAGCAGAGAGCACAGA |
| IL-1β R | GGGTTCCATGGTGAAGTCAACT |
| IL-1α F | GGAGGCCATAGCCCATGATTT |
| IL-1α R | TGAACTCCTGCTTGACGATCC |
| TNF-α F | AGATGTGGAACTGGCAGAGG |
| TNF-α R | CCCATTTGGGAACTTCTCCT |
| GM-CSF F | AAGCAGGGTCTACGGGGCAA |
| GM-CSF R | CTCATTTCTGGACCGGCTTC |
| IFN-γ F | TCGCACCTGATCACTAACTTCTTC |
| IFN-γ R | GTGGGTTGTTCACCTCGAACTT |
| CCL2 F | GGTCTCTGTCACGCTTCTG |
| CCL2 R | TTCTCCAGCCGACTCATTG |
| CCL5 F | CACCTGCCTCCCCATATG |
| CCL5 R | TTCCTTCGAGTGACAAAGACG |
| GAPDH F | ACCACAGTCCATGCCATCAC |
| GAPDH R | TCCACCACCCTGTTGCTGTA |
| MYH6 F | CCATCTCTGACAACGCCTATC |
| MYH6 R | TCTCCAGAAGGTAGGTCTCTATG |
| ATP2A2 F | GTATGACGGGCTTGTAGAGTTAG |
| ATP2A2 R | CGATCTTAGACTTGTGGGAAGG |

# Supplementary Figures and Tables

**Table S1**. Physicochemical characterization of Rho-T3-lipo. Sizes, polydispersity index (PDI), Zeta potential, encapsulation efficiency (EE) and drug loading (DL) of Rho-T3-lipo. Mean ±SD values were obtained from at least three independent experiments performed in triplicate.

|  | Size(nm) | PDI | Zeta potential(mV) | EE（%） | DL（%） |
| --- | --- | --- | --- | --- | --- |
| Rho-T3-lipo | 203.37±5.92 | 0.21±0.031 | 5.37±0.67 | 85.83±0.087 | 8.37±0.0085 |

**Supplementary Figure 1.** Inhibitory effect of T3-lipo on hepatocarcinogenesis in DEN-induced primary HCC rat models. (A) Experimental design. (B) Representative upper and visceral views of livers from rats treated with saline, T3, and T3-lipo at a dose of 0.5mg/kg. (n=4) (C)Tumor incidence (%). (D)Number of tumors (n). (E)Max volume of tumors (mm³). (F) H&E staining of liver tissues. Scale bars represent 200μm. (G) Serum levels of CK(U/L). (H) Serum levels of CK-MB(U/L). n=4 rats per group. Three independent experiments were performed for all data. Differences were analyzed using One-way ANOVA. (*p < 0.05, **p < 0.01, ***p < 0.001, ****p < 0.0001, N.S., not significant).


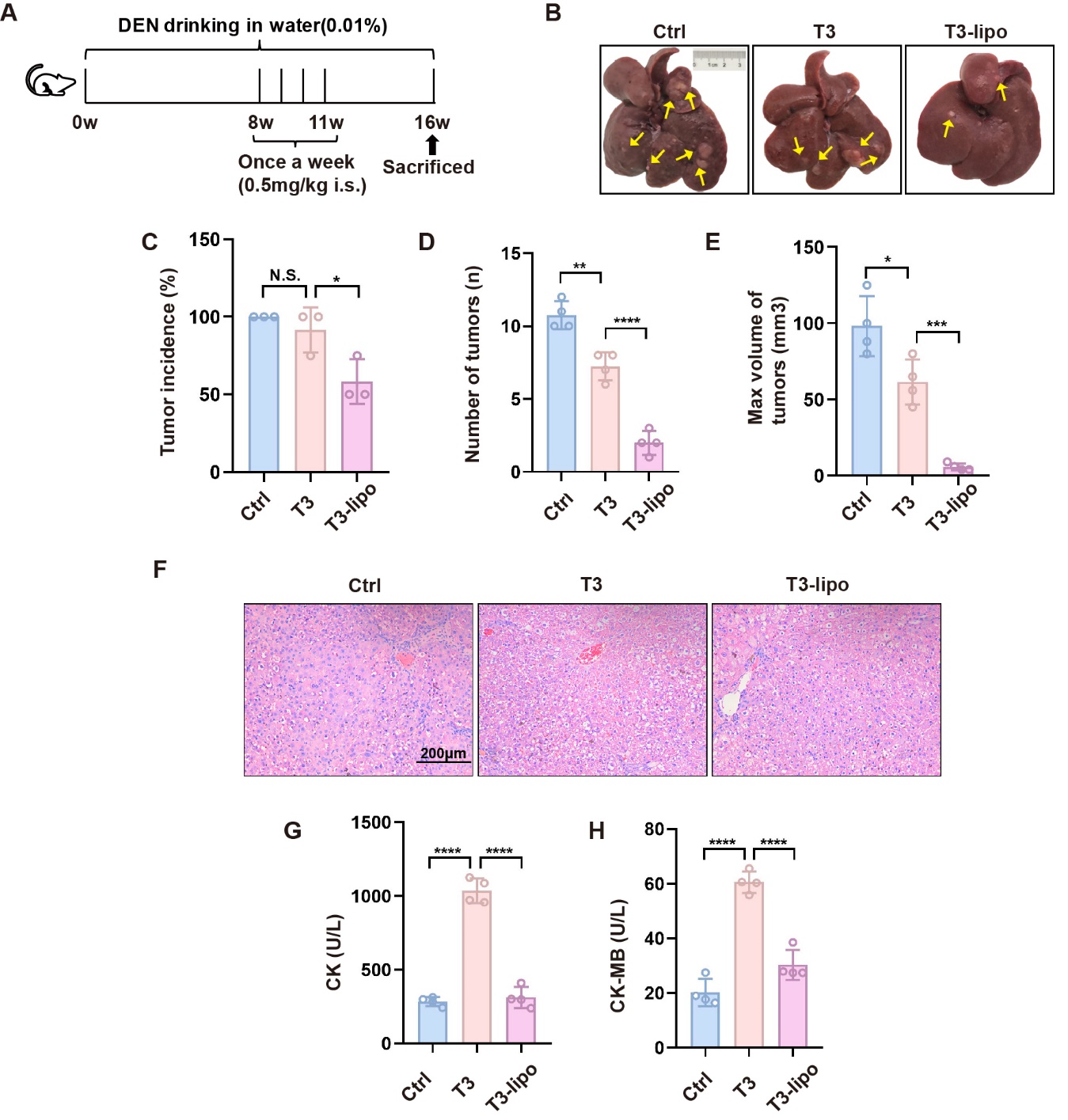


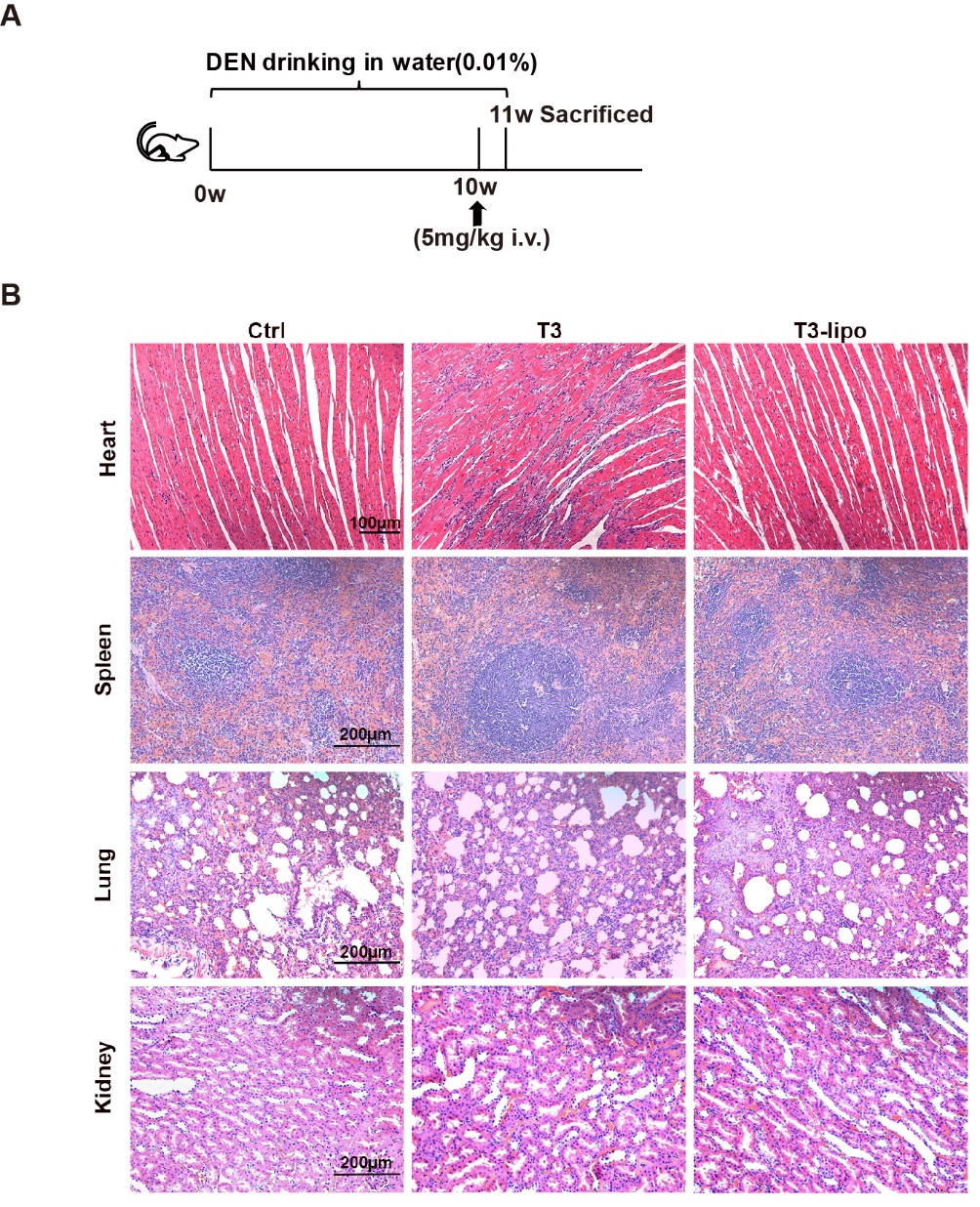
**Supplementary Figure 2.** The histological characteristics of other organs after treatment with saline, T3, and T3-lipo. (A) Experimental design. (B) H&E staining of heart, spleen, lung, and kidney tissues.

**Supplementary Figure 3.** T3-lipo affect the polarization of macrophages. (A) IHC staining of CD68, iNOS, and CD163 in peritumoral tissues from 11 weeks DEN-induced rats with different ways of administration (5mg/kg, *i.v.*). (B) Quantification of the CD68, iNOS, and CD163 positive cell numbers. Mean ±SD values were obtained from at least three independent experiments performed in triplicate. Differences were analyzed using One-way ANOVA. (*p < 0.05, **p < 0.01, N.S., not significant)


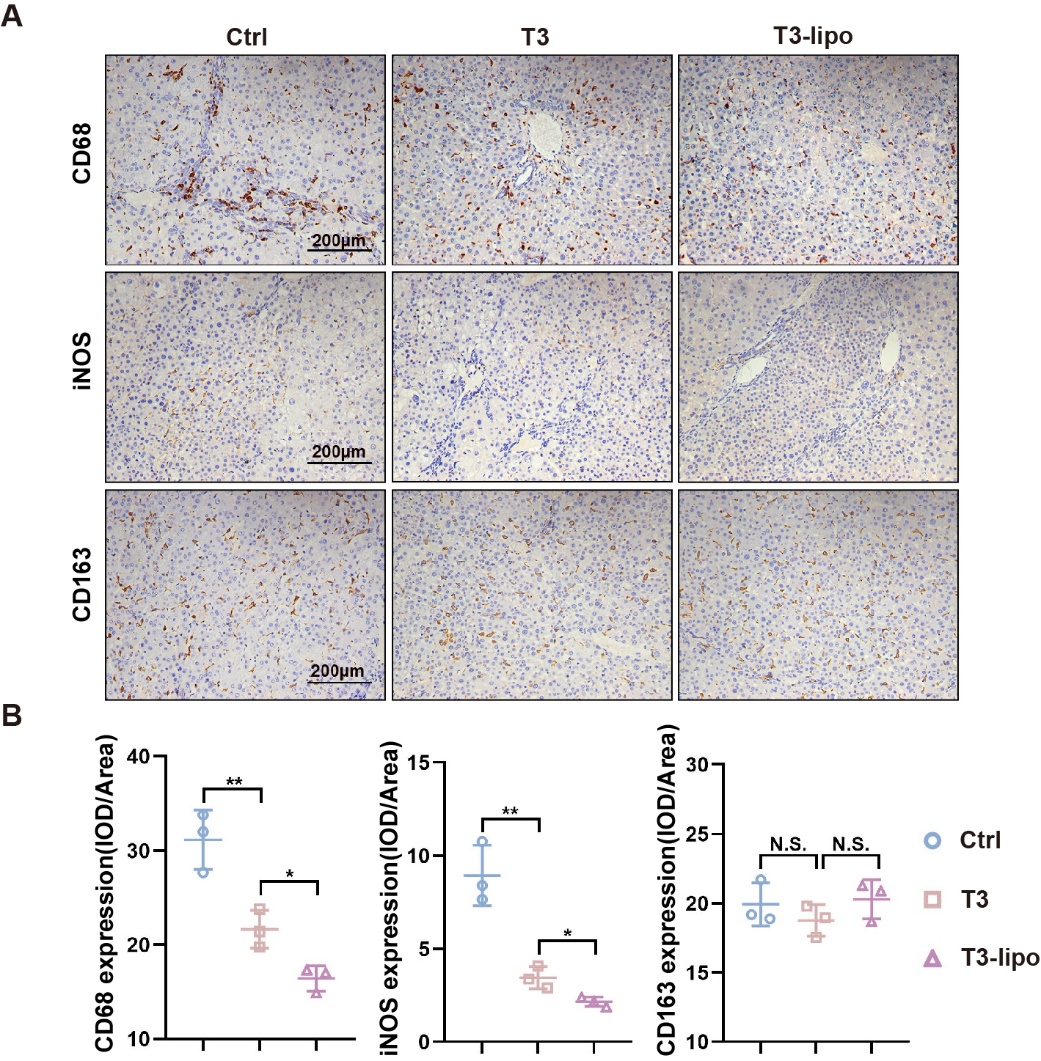

Supplement: Supplementary file 1 [file DataSheet_1.docx]
